# Discovery of cancer vaccination protocols with a genetic algorithm driving an agent based simulator

**DOI:** 10.1186/1471-2105-7-352

**Published:** 2006-07-20

**Authors:** Pier-Luigi Lollini, Santo Motta, Francesco Pappalardo

**Affiliations:** 1 Sezione di Cancerologia, Dipartimento di Patologia Sperimentale, University of Bologna, and Centro Interdipartimentale di Ricerche sul Cancro "Giorgio Prodi", Italy; 2 Department of Mathematics and Computer Science, University of Catania, Catania, Italy; 3 Faculty of Pharmacy, University of Catania, Italy

## Abstract

**Background:**

Immunological prevention of cancer has been obtained in HER-2/neu transgenic mice using a vaccine that combines 3 different immune stimuli (Triplex vaccine) that is repeatedly administered for the entire lifespan of the host (Chronic protocol). Biological experiments leave open the question of whether the Chronic protocol is indeed the minimal vaccination schedule affording 100% protection, or whether shorter protocols could be applied that would result in the same efficacy. A biological solution would require an enormous number of experiments, each lasting at least one year. Therefore we approached this problem by developing a simulator (SimTriplex) which describes the immune response activated by Triplex vaccine. This simulator, tested against *in vivo *experiments on HER-2/neu mice, reproduces all the vaccination protocols used in the *in vivo *experiments. The simulator should describe any vaccination protocol within the tested range. A possible solution to the former open question using a minimal search strategy based on a genetic algorithm is presented. This is the first step toward a more general approach of biological or clinical constraints for the search of an effective vaccination schedule.

**Results:**

The results suggest that the Chronic protocol included a good number of redundant vaccine administrations, and that maximal protection could still be obtained with a number of vaccinations ~40% less than with the Chronic protocol.

**Conclusion:**

This approach may have important connotations with regard to translation of cancer immunopreventive approaches to human situations, in which it is desirable to minimize the number of vaccinations. We are currently setting up experiments in mice to test whether the actual effectiveness of the vaccination protocol agrees with the genetic algorithm.

## Background

Experiments in transgenic mice showed that mammary carcinogenesis driven by the HER-2/neu oncogene can be completely prevented by prophylactic vaccines that elicit protective immune responses [1,2]. One of the most effective vaccines was made of cells expressing the HER-2/neu antigen and two adjuvant signals, interleukin 12 (IL-12) and allogeneic class I major histocompatibility complex antigens, referred to as the Triplex vaccine [1,3].

A complete prevention of mammary carcinogenesis with the Triplex vaccine was obtained when vaccination cycles (one intraperitoneal vaccination every 3–4 days for a total of 4 vaccinations over 2 weeks, followed by 2 weeks of rest) started at 6 weeks of age and continued for the entire duration of the experiment, at least 1 year (Chronic protocol). Various attempts at reducing *in vivo *the number of vaccinations invariably resulted in all mice succumbing to tumors. This was the case with experiments in which we tested the effects of just 3 vaccination cycles starting at 6, 10 or 16 weeks of age (Early, Late and Very late protocols, respectively) [1].

Biological experiments leave open the question of whether the Chronic protocol is indeed the minimal vaccination schedule affording 100% protection, or whether shorter protocols could be applied that would result in the same efficacy. A biological solution would require an enormous number of experiments, each lasting at least a year. We approached this problem in two steps. First we developed a mathematical model/simulator (SimTriplex) which describes the immune response activated by the Triplex vaccine and we validated it using all existing *in vivo *experiments, then we used the simulator to search *in silico *for optimal vaccination protocols, defined here as protocols that minimize the number of vaccinations without reducing tumor prevention efficacy in comparison to the Chronic protocol.

Mathematical and computer models of the immune response have been proposed over the last two decades, using a variety of different approaches. The model we use to describe the cancer-immune system competition induced by the Triplex vaccine originates from the cellular automata-like approach proposed by Celada and Seiden [4].

**Figure 1:**
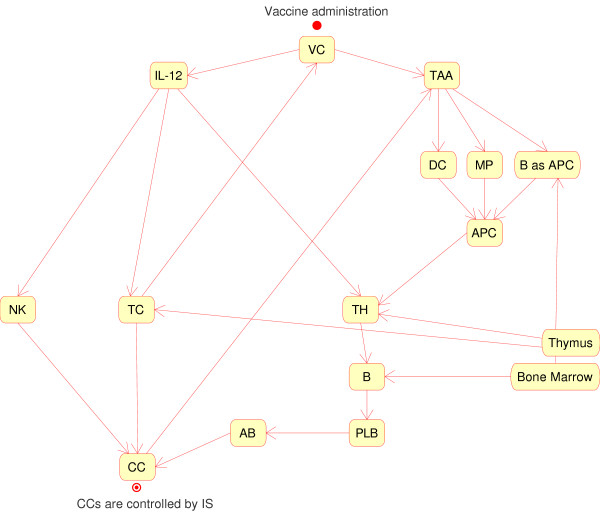
**SimTriplex logic interactions chart**. Vaccine Cells (VC) produce interleukin 12 (IL-12) and release Tumor Associated Antigens (TAA) when they die either spontaneously or as a consequence of cytotoxic T cell (TC) lysis upon recognition of allogeneic MHC on VC. TAA interact with antigen presenting cells (APC), i.e. macrophages (MP), dendritic cells (DC) and B cells, which internalize TAA and become able to stimulate helper T cells (TH). A TH↔B interaction induces differentiation of B cells into plasma cells (PLB) which produce antibodies (Ab) recognizing cancer cells (CC). IL-12 enhances the effectiveness of Natural Killer (NK) and TC against CC. The final state is that CC are controlled by immune responses activated by VC. All the interactions embed a probabilistic rule which depends on biological affinity or other factors.

Using this approach, one can describe all the relevant entities of the phenomena and their interactions by means of rules derived from biological experiences. In our model and simulator we describe the most relevant entities and processes (immune system, cancer cells, vaccine cells) needed to reproduce the immune response induced by the vaccine, a detailed description being found in the references [5] and [6], Figure 1 summarizes the main logical interactions in the SimTriplex simulator.

As reported, the model and the simulator have been validated against existing *in vivo *experiments. *In silico *experiments show excellent agreement with *in vivo *experiments, and in the validation range SimTriplex represents a good cancer-immune system simulator [6].

A validated simulator will reasonably reproduce, in the validation range, the immune response activated by a vaccination protocol, thus one can reproduce *in silico *different vaccination schedules and search for the "best" ones, i.e. the schedules with the minimum number of vaccine administrations which still prevent tumor formation (optimal schedule).

In searching for an optimal schedule, we have tried different strategies. The first attempt was made by a "trial and error" method. We set successively repeating cycles of injections at different stages of the virtual mouse age, and the simulator was used to determine the survival of vaccinated mice. In this way we found an effective schedule of only 44 vaccinations, that is 27% less than the standard Chronic protocol [5]. A second search strategy was based on genetic algorithms [7]. Attempts at using an unconstrained genetic algorithm led to the conclusion that a genetic search should be constrained on biological bases [8]. Furthermore it must be kept in mind that *in vivo *experiments in transgenic mice, as all biological experiments, are affected by natural immunological variability resulting from subtle individual variations in the generation of the immunological repertoire, and in interactions with environmental variables [3]. The SimTriplex simulator, and its ancestor ImmSim [4], faithfully model this aspect, for example, through the generation of a random repertoire of antigen receptors in each *in silico *mouse. From previous experience [8], we concluded that a genetic search should take into account simultaneously different simulated individuals and we present what is probably a satisfactory result. Section Results provides computational results. And in Section Conclusions, the conclusions and final remarks are made.

## Results

On the parallel machine the genetic algorithm required 72 h and returned a 35 injections schedule. This schedule was then applied to the 2 samples of 100 mice and estimated that 88% of the mice remained tumor-free at 400 days of age. Figure 3 shows that the tumor-free survival curves of groups of two samples of 100 virtual mice and 8 actual mice vaccinated according to different protocols were almost identical. Figures 4–6 show the evolution of the mean values of the relevant immune responses when the GA vaccination schedule was applied to the mice in the sample (left column graphs, labeled GA-s) versus the same quantities previously computed [6] for the Chronic schedule (right column graphs, labeled CH-s). As shown in [5], it should be remembered that the error level in steady phase of Chronic schedule plots (CH-s) was 5–8%.

The cancer cell plot (Figure 4) includes lines showing the upper and lower limits of the error, as well a top straight line which represents the limit of solid tumor formation and a stepwise line which represents the limits imposed to the GA search (equation 6).

First, note that the cancer cells plot for CH-s is almost flat for *t *> 200 days, while the plot for GA-s shows an increasing number of cancer cells. The latter behavior is consistent with the imposed constraints requirement, for *t *> t*, to the GA, which was a safer level. This means that CH-s includes many redundant vaccine injections.

The same effect could be seen in tumor-associated antigens behavior (Figure 4). This is consistent with the cancer cells plots, since a higher number of depleted cancer cells would produce a higher number of TAA. Helper T cell behavior in GA-s (Figure 5) is almost equal (± 1%), i.e. inside the errors bar, to the CH-s one. It is interesting to compare the plots of cytotoxic T-cells. The GA-s plots show in the initial phase (*t *≤ 150 days), a smaller value of the peak which is outside the error bar limits. This means that the new schedule would produce a smaller cytotoxic response. In the second phase (*t *≤ 150 days), the new schedule shows two small humps (both outside the error limits) which are due to the cytoxic response to the cancer cells peaking in the same period.

The GA-s B-cell plot (Figure 6) shows again slightly larger oscillation in the second period (*t *> 150 days). Those oscillations are effective (i.e. outside error bars) and due, as before, to secondary TAA growth. The antibodies plots for both schedules (Figure 6) are equal in the limits of the error bars. This suggests that humoral response will be able to control the tumor growth in the new schedule as in the Chronic one.

## Discussion

We have presented an evolutionary algorithm which efficiently finds effective vaccination schedules for protecting virtual mice from mammary carcinoma. Modeling immune response has been attacked over the last two decades [4,9]. However, as far we know, this is the first attempt to use a validated simulator to predict immune response stimulated by a vaccine. The model prediction, if confirmed by our *in vivo *experiments, has practical application in vaccine discovering and testing.

Comparing the behaviors of the relevant biological and immunological responses in the 2 schedules shows that the new protocol controls the tumor growth in much the same way as the Chronic schedule. The genetic algorithm outlined here is the most efficient method tested so far to find optimal vaccination schedules in this biological model system. An alternative schedule has been suggested in [5], based on a "trial and error" method (Figure 10 and 11 in [5]). Comparison of this result with the one proposed in Figure 3 shows that the GA search is more effective than the trial-and-error one because it found a schedule of equal potency with less vaccinations.

The design of vaccination schedules is a key element in determining the protective effect of a vaccine [10]. However most actual schedules are decided a priori on a purely empirical basis, with a few "stepping stones" derived from basic immunological knowledge. Only after a sufficient number of individuals has been vaccinated is it possible to define immunological correlates of protection (e.g. serum antibody titers) that can be used to guide, once again empirical, refinements of the vaccination protocol [11]. The latter approach works better for vaccines against infectious agents than cancer because immunological parameters measured in peripheral blood correlate poorly with the immune response inside neoplastic lesions [12,13]. Paradoxically it is easier to improve on poorly effective schedules rather than to optimize effective ones, because once a successful protocol is established one does not risk a loss of protection just to spare some vaccinations, especially if side effects of repeated vaccinations are of minor consequence. The problem of defining optimal schedules was particularly acute in cancer immunopreventive approaches, like the Triplex vaccine, which must keep a high level of protective immunity against a continuing generation of cancer cells for very long periods, ideally for the entire lifetime of the host [2]. Experimental evidence showed that vaccination protocols much shorter than the Chronic one only resulted in a delay of mammary carcinogenesis, but all mice eventually succumbed to tumors [1]. However, the very long duration of experiments (at least one year), combined with the high number of vaccinations actually forbade an exhaustive search of a minimal vaccination protocol.

## Conclusion

The results of the genetic algorithm applied to the SimTriplex simulator suggest that the Chronic protocol included a good number of redundant vaccine administrations, and that maximal protection could still be obtained by halving the number of vaccinations. This is an important result with regard to translation of cancer immunopreventive approaches to human situations, in which it is desirable to keep the number of vaccinations to a minimum, and *in vivo *experiments in mice to test the actual effectiveness of the vaccination protocol indicated by the genetic algorithm are now being set up.

The possible outcomes of the experimental validation include complete protection from tumor onset, indicating that further reduction in the number of vaccinations is feasible; or a significantly low degree of protection, indicating that more vaccinations are required. On the basis of the results of validation experiments, we will iteratively implement a cyclical refinement of the computer model to define further biological experiments, a strategy that was shown to significantly improve the efficiency of research [14,15].

## Methods

**Figure 2:**
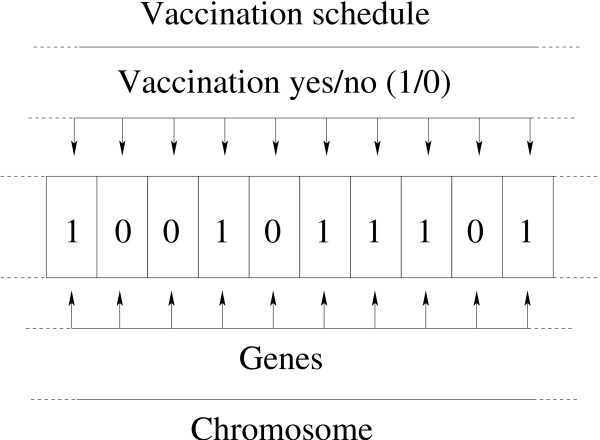
**Correspondence between vaccinations and the elements of a genetic algorithm**. A complete vaccination schedule was encoded into a bitstring. Each bit represents a 8 h time-step in which a single vaccination can be administered (bit = 1) or not (bit = 0). In genetic algorithm parlance each bit is a gene and the entire bitstring a chromosome.

**Figure 3:**
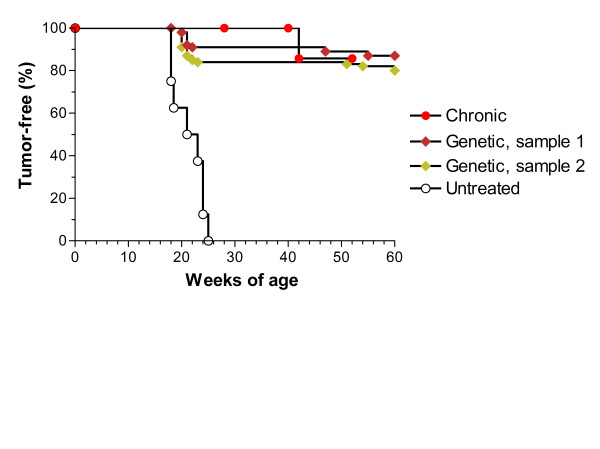
**Tumor-free survival curves**. Tumor-free survival curves of groups of virtual and actual mice vaccinated according to different protocols. " Untreated" and " Chronic", groups of 8 actual mice receiving either no treatment or vaccinations according to the Chronic protocol, respectively [3]. "Genetic, sample 1" and "Genetic, sample 2": groups of 100 virtual mice vaccinated according to the protocol yielded by the genetic algorithm.

**Figure 4:**
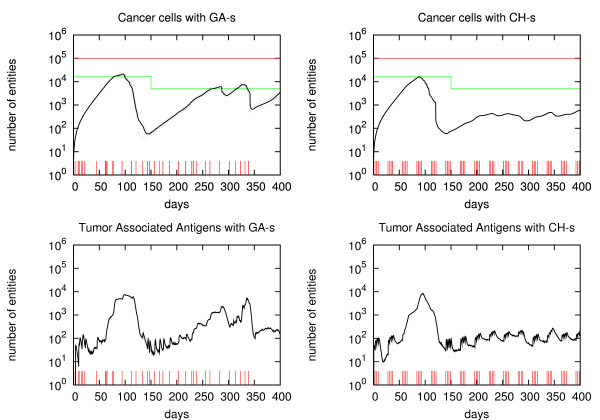
**Kinetics of cancer cells and tumor associated antigens**. The number of CC and TAA due to vaccinations according to the GA schedule (GA-s, left panels) and Chronic schedule (CH-s, right panels) is shown versus time in days. Small vertical lines indicate the vaccine's injection times.

**Figure 5:**
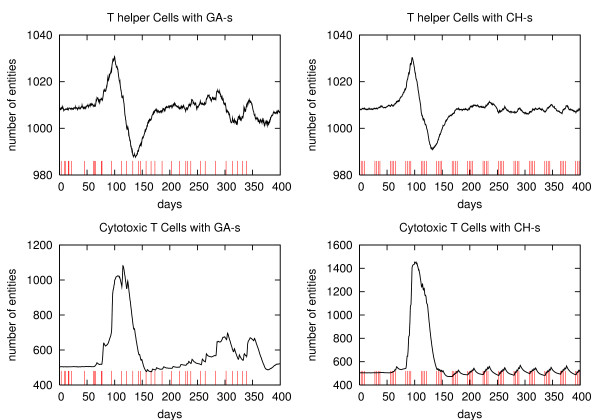
**Kinetics of T cell responses**. Number of helper T cells and cytotoxic T cells during vaccinations according to the GA schedule (GA-s, left panels) and Chronic schedule (CH-s, right panels) is shown versus time in days. Small vertical lines indicate the vaccine's injection times.

**Figure 6:**
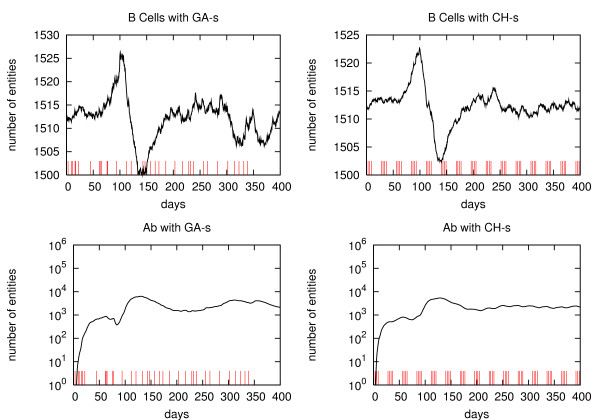
**Kinetics of humoral immune response**. Activation of humoral immune responses due to the GA schedule (GA-s, left panels) and Chronic schedule (CH-s, right panels) is shown versus time in days. Small vertical lines indicate the vaccine's injection times.

Standard theory of Genetic Algorithms (GA) was first introduced by Holland in 1975 [7]. Our approach differs from a standard GA since it uses a simulator to compute the fitness function. To the best of our knowledge, very few examples of this type exist in the literature and none in bioinformatics. First, the entities of the GA are defined according to the usual terminology of GA literature [7]. Each GA's chromosome in the chromosomes' population represents a vaccine schedule (Figure 2). The chromosome is a binary string of 1200 bits, in which each gene (i.e. each bit) represents a time-step, *t*_*i*_, during which it is possible to inject a vaccine dose. The time interval *δt *= *t*_*i*+1 _- *t*_*i *_is a constant and it is ~ 8 h of actual time. If the *i*-th gene is expressed, i.e. the *i*-th bit is set to 1, then a vaccination has to be administered at time-step *i*; otherwise if the *i*-th gene is not expressed, i.e. the *i*-th bit is set to 0, then no vaccination has to be administered at time-step *i*. The set comprises 80 chromosomes.

The **selection operator **used is *tournament selection *[16]. **Reproduction **uses uniform crossover; **mutation and elitism **were implemented in a standard way [7].

SimTriplex simulator computes the main biological entities of the cancer – immune system competition. If the number of cancer cells is > 10^5^, then the simulator recognizes the solid tumor formation (carcinogenesis) and simulation ends at the time that has been reached. We will refer to this time as the mouse survival time. An effective vaccination must reach a mouse survival time of 1200 time-steps equal to a lifespan of 400 days.

In defining the **fitness function **we must take into account 2 fundamental and competing requirements: *i) *any schedule must be an effective one, i.e. the mouse survival time must reach 400 days; *ii) *the best schedules must have a minimal cardinality, i.e. they must provide mice survival with the minimum number of vaccine injections.

Any evolutionary approach which only takes into consideration the first requirement would produce chromosomes very rich of 1, thus not minimal. If instead we take into consideration just the second requirement, we would get chromosomes full of 0, and thus very likely we would obtain a non-effective schedule.

Therefore the fitness function must be at least a 2 variable function of type *f *(*n, s*, ...), where *n *is the number of injections, and *s *is the mouse survival time measured in time-steps; it must be a decreasing function with respect to number of injections and an increasing function with respect to survival time. The following 2 properties must hold:

*f *(*n, s*,…) <*f *(*n, s*',…) iff *s *> *s' *    (1)

*f *(*n, s*,…) > *f *(*n', s*,…) iff *n *> n'     (2)

The simplest example of a 2 variable fitness function is *f *(*n, s*) = *n*^2^/*s*. Tests using this fitness function on a single mouse [8] yielded very high peaks in cancer cell number. Those peaks were below, but very close to, the threshold of solid tumor formation. Even if a solid tumor is not yet formed, a high number of cancer cells may induce, by overstimulation, an anergic state of T lymphocytes, depleting in this way the immune system response and enhancing the risk of carcinogenesis. We concluded that it is better to include a control on tumor growth in the fitness function, to reproduce the behavior of cancer cells in the simulation of the Chronic protocol, which effectively prevented tumors in mice [6]. For this we chose a 3 variable function for a single mouse, namely:

f(n,s,β)=n2s⋅β     (3)
 MathType@MTEF@5@5@+=feaafiart1ev1aaatCvAUfKttLearuWrP9MDH5MBPbIqV92AaeXatLxBI9gBaebbnrfifHhDYfgasaacH8akY=wiFfYdH8Gipec8Eeeu0xXdbba9frFj0=OqFfea0dXdd9vqai=hGuQ8kuc9pgc9s8qqaq=dirpe0xb9q8qiLsFr0=vr0=vr0dc8meaabaqaciaacaGaaeqabaqabeGadaaakeaacqWGMbGzcqGGOaakcqWGUbGBcqGGSaalcqWGZbWCcqGGSaaliiGacqWFYoGycqGGPaqkcqGH9aqpdaWcaaqaaiabd6gaUnaaCaaaleqabaGaeGOmaidaaaGcbaGaem4CamhaaiabgwSixlab=j7aIjaaxMaacaWLjaWaaeWaceaacqaIZaWmaiaawIcacaGLPaaaaaa@42AB@

where *β *is defined as:

β={Ncc1γ1+Ncc2γ2if Ncc1≥γ1∨Ncc2≥γ21.0otherwise     (4)
 MathType@MTEF@5@5@+=feaafiart1ev1aaatCvAUfKttLearuWrP9MDH5MBPbIqV92AaeXatLxBI9gBaebbnrfifHhDYfgasaacH8akY=wiFfYdH8Gipec8Eeeu0xXdbba9frFj0=OqFfea0dXdd9vqai=hGuQ8kuc9pgc9s8qqaq=dirpe0xb9q8qiLsFr0=vr0=vr0dc8meaabaqaciaacaGaaeqabaqabeGadaaakeaaiiGacqWFYoGycqGH9aqpdaGabeqaauaabaqaciaaaeaadaWcaaqaaiabd6eaonaaDaaaleaacqWGJbWycqWGJbWyaeaacqaIXaqmaaaakeaacqWFZoWzdaWgaaWcbaGaeGymaedabeaaaaGccqGHRaWkdaWcaaqaaiabd6eaonaaDaaaleaacqWGJbWycqWGJbWyaeaacqaIYaGmaaaakeaacqaHZoWzdaWgaaWcbaGaeGOmaidabeaaaaaakeaacqqGPbqAcqqGMbGzcqqGGaaicqWGobGtdaqhaaWcbaGaem4yamMaem4yamgabaGaeGymaedaaOGaeyyzImRae83SdC2aaSbaaSqaaiabigdaXaqabaGccqGHOiI2cqWGobGtdaqhaaWcbaGaem4yamMaem4yamgabaGaeGOmaidaaOGaeyyzImRae83SdC2aaSbaaSqaaiabikdaYaqabaaakeaacqaIXaqmcqGGUaGlcqaIWaamaeaacqWGVbWBcqWG0baDcqWGObaAcqWGLbqzcqWGYbGCcqWG3bWDcqWGPbqAcqWGZbWCcqWGLbqzaaaacaGL7baacaWLjaGaaCzcamaabmGabaGaeGinaqdacaGLOaGaayzkaaaaaa@6C16@

and Ncc1
 MathType@MTEF@5@5@+=feaafiart1ev1aaatCvAUfKttLearuWrP9MDH5MBPbIqV92AaeXatLxBI9gBaebbnrfifHhDYfgasaacH8akY=wiFfYdH8Gipec8Eeeu0xXdbba9frFj0=OqFfea0dXdd9vqai=hGuQ8kuc9pgc9s8qqaq=dirpe0xb9q8qiLsFr0=vr0=vr0dc8meaabaqaciaacaGaaeqabaqabeGadaaakeaacqWGobGtdaqhaaWcbaGaem4yamMaem4yamgabaGaeGymaedaaaaa@318C@ is the maximum number of cancer cells in the lattice during the time interval [0, t*]; and Ncc2
 MathType@MTEF@5@5@+=feaafiart1ev1aaatCvAUfKttLearuWrP9MDH5MBPbIqV92AaeXatLxBI9gBaebbnrfifHhDYfgasaacH8akY=wiFfYdH8Gipec8Eeeu0xXdbba9frFj0=OqFfea0dXdd9vqai=hGuQ8kuc9pgc9s8qqaq=dirpe0xb9q8qiLsFr0=vr0=vr0dc8meaabaqaciaacaGaaeqabaqabeGadaaakeaacqWGobGtdaqhaaWcbaGaem4yamMaem4yamgabaGaeGOmaidaaaaa@318E@ is the same quantity in the time interval [t*, 1200]. t* has been chosen to be equal to 150 time-steps in order to distinguish the transient phase from the steady one [8]. Finally we chose the 2 constants as follows: *i) γ*_1 _= 1.7·10^4 ^(the height of the first peak in the simulation of the Chronic protocol [6]); *ii) γ*_2 _= 5·10^3 ^(slightly higher than in the Chronic protocol but well below the tumor threshold). Fitness function (3) meets the requested properties (1) and (2).

The GA found a schedule that maintains a cancer cell threshold below the requested one in the test individual, but the same schedule applied to the large statistical sample previously used [8] returned 20% tumor-free mice. This was an unsatisfactory, but expected, result as the mice in the sample reproduce a large class of different *phenotypes *that encompasses biological variability originating from individual variations in the immunological repertoire of clonotypic T and B cell receptors, and in postnatal interactions between the immune system and the environment.

To find an effective protocol for a larger proportion of mice in the sample, we applied the same strategy to 8 different instances of *in silico *mice using different random seeds for the generation of the repertoire of bitstrings that are used by SimTriplex to simulate the repertoire of T and B antigen receptors. The fitness function (3) was modified to take into account all the chosen mice simultaneously, which was simply obtained by summing up the fitness function for each mouse:

fg(n,s1,...,s8,β1,...,β8)=∑i=18f(n,si,βi)     (5)
 MathType@MTEF@5@5@+=feaafiart1ev1aaatCvAUfKttLearuWrP9MDH5MBPbIqV92AaeXatLxBI9gBaebbnrfifHhDYfgasaacH8akY=wiFfYdH8Gipec8Eeeu0xXdbba9frFj0=OqFfea0dXdd9vqai=hGuQ8kuc9pgc9s8qqaq=dirpe0xb9q8qiLsFr0=vr0=vr0dc8meaabaqaciaacaGaaeqabaqabeGadaaakeaacqWGMbGzdaWgaaWcbaGaem4zaCgabeaakiabcIcaOiabd6gaUjabcYcaSiabdohaZnaaBaaaleaacqaIXaqmaeqaaOGaeiilaWIaeiOla4IaeiOla4IaeiOla4IaeiilaWIaem4Cam3aaSbaaSqaaiabiIda4aqabaGccqGGSaaliiGacqWFYoGydaWgaaWcbaGaeGymaedabeaakiabcYcaSiabc6caUiabc6caUiabc6caUiabcYcaSiab=j7aInaaBaaaleaacqaI4aaoaeqaaOGaeiykaKIaeyypa0ZaaabCaeaacqWGMbGzcqGGOaakcqWGUbGBcqGGSaalcqWGZbWCdaWgaaWcbaGaemyAaKgabeaakiabcYcaSiab=j7aInaaBaaaleaacqWGPbqAaeqaaOGaeiykaKcaleaacqWGPbqAcqGH9aqpcqaIXaqmaeaacqaI4aaoa0GaeyyeIuoakiaaxMaacaWLjaWaaeWaceaacqaI1aqnaiaawIcacaGLPaaaaaa@5FD4@

where *s*_*i *_is the survival time of mouse *i*; *β*_*i *_is defined as:

βi={Ncci1γ1+Ncci2γ2if Ncci1≥γ1∨Ncci2≥γ21.0otherwise     (6)
 MathType@MTEF@5@5@+=feaafiart1ev1aaatCvAUfKttLearuWrP9MDH5MBPbIqV92AaeXatLxBI9gBaebbnrfifHhDYfgasaacH8akY=wiFfYdH8Gipec8Eeeu0xXdbba9frFj0=OqFfea0dXdd9vqai=hGuQ8kuc9pgc9s8qqaq=dirpe0xb9q8qiLsFr0=vr0=vr0dc8meaabaqaciaacaGaaeqabaqabeGadaaakeaaiiGacqWFYoGydaWgaaWcbaacbiGae4xAaKgabeaakiabg2da9maaceqabaqbaeaabiGaaaqaamaalaaabaGaemOta40aa0baaSqaaiabdogaJjabdogaJnaaBaaameaacqGFPbqAaeqaaaWcbaGaeGymaedaaaGcbaGae83SdC2aaSbaaSqaaiabigdaXaqabaaaaOGaey4kaSYaaSaaaeaacqWGobGtdaqhaaWcbaGaem4yamMaem4yam2aaSbaaWqaaiabdMgaPbqabaaaleaacqaIYaGmaaaakeaacqWFZoWzdaWgaaWcbaGaeGOmaidabeaaaaaakeaacqqGPbqAcqqGMbGzcqqGGaaicqWGobGtdaqhaaWcbaGaem4yamMaem4yam2aaSbaaWqaaiabdMgaPbqabaaaleaacqaIXaqmaaGccqGHLjYScqWFZoWzdaWgaaWcbaGaeGymaedabeaakiabgIIiAlabd6eaonaaDaaaleaacqWGJbWycqWGJbWydaWgaaadbaGaemyAaKgabeaaaSqaaiabikdaYaaakiabgwMiZkab=n7aNnaaBaaaleaacqaIYaGmaeqaaaGcbaGaeGymaeJaeiOla4IaeGimaadabaGaem4Ba8MaemiDaqNaemiAaGMaemyzauMaemOCaiNaem4DaCNaemyAaKMaem4CamNaemyzaugaaaGaay5EaaGaaCzcaiaaxMaadaqadiqaaiabiAda2aGaayjkaiaawMcaaaaa@73F3@

A GA with an attached simulator is a long computational task, with a single mouse run taking 36 h on a Pentium class machine. The fitness function described above requires a prohibitive amount of running time on a single CPU machine. We rewrote our GA in a parallel programming language (MPI) and launched it on a 32-nodes parallel cluster machine.

## Authors' contributions

PLL contributed the immunological and oncological expertise for the development of the simulator and of the genetic algorithm, and provided results of *in vivo *vaccination experiments.

SM conceived the application of an immunological simulator to cancer vaccines, including the use of genetic algorithms for the discovery of vaccination protocols, supervised the whole project and drafted the manuscript.

FP provided expertise in genetic algorithm, he first suggested and then developed the software for the genetic algorithm, including an ad hoc version of the simulator and its implementation on parallel machines.

All authors read and approved the final manuscript.
